# Early Metabolic and Oxidative Effects of Hop Extract, Alendronate, and Their Combination Across Tissues in an Estrogen-Deficient Rat Model: An Exploratory Study

**DOI:** 10.3390/nu18111685

**Published:** 2026-05-25

**Authors:** Edi Rođak, Nika Srb, Robert Grgac, Željko Debeljak, Ivana Ilić, Nada Oršolić, Nikola Bijelić

**Affiliations:** 1 Department of Histology and Embryology, Faculty of Medicine Osijek, University of Osijek, J. Huttlera 4, 31000 Osijek, Croatia; erodak@mefos.hr (E.R.); nsrb@mefos.hr (N.S.); rgrgac@mefos.hr (R.G.); iilic@fdmz.hr (I.I.); 2 Department of Medicine, University of Connecticut Health Centre, 263 Farmington Ave., Farmington, CT 06030, USA; 3 Department of Clinical Laboratory Diagnostics, University Hospital Centre Osijek, J. Huttlera 4, 31000 Osijek, Croatia; zdebeljak@mefos.hr; 4 Department of Pharmacology, Faculty of Medicine Osijek, University of Osijek, J. Huttlera 4, 31000 Osijek, Croatia; 5 Department of Anatomy, Histology, Embryology, Pathological Anatomy and Pathological Histology, Faculty of Dental Medicine and Health, University of Osijek, Crkvena 21, 31000 Osijek, Croatia; 6 Department of Animal Physiology, Faculty of Science Zagreb, University of Zagreb, Rooseveltov trg 6, 10000 Zagreb, Croatia; nada.orsolic@biol.pmf.hr

**Keywords:** alendronate, estrogen, hop extract, metabolism, osteoporosis, oxidative stress, serum lipids, rat

## Abstract

Background/Objectives: Postmenopausal osteoporosis is associated with systemic metabolic alterations related to estrogen deficiency. This exploratory study investigated the early metabolic, oxidative, and histomorphological effects of alendronate, a standardized hop extract, and their combination in ovariectomized (OV) rats. Methods: Female Wistar rats (*n* = 70) were randomly allocated to seven groups: six OV groups or a sham-operated control (*n* = 10 per group). Following a 30-day postoperative period for model development, animals received daily treatment for 2 weeks with vehicle (placebo), low- or high-dose alendronate (1 and 2 mg/kg, respectively), standardized hop extract (60 mg/kg), or both. Oxidative stress markers, liver and perigonadal adipose tissue histology, and tissue metabolism were assessed. Results: No evidence of adverse hepatic, renal, or systemic effects was observed. Oxidative damage markers remained largely unchanged, although ovariectomy was associated with reduced hepatic catalase activity, which was increased by high-dose alendronate. Treatment-related morphological changes in adipose tissue were observed. Serum triglyceride levels were unaffected, whereas total cholesterol was significantly increased in animals receiving hop extract. Hepatic triglyceride levels were influenced by alendronate treatment, modified by hop extract. MALDI-TOF MS suggested no OV-related alterations in amino acid, lipid, steroid, and redox-related metabolism in liver and adipose tissue. The subsequent treatments, especially in the high-dose alendronate group and hop-extract group, partially modified these metabolic signatures; however, these findings remain provisional pending MS/MS validation. Conclusions: Most outcomes were not significantly altered by OV. Alendronate and hop extract exert distinct short-term effects on selected metabolic and oxidative parameters in this exploratory model. Further studies are needed to investigate translational potential.

## 1. Introduction

Osteoporosis is a multifactorial disorder characterized by reduced bone mineral density and is classified as either primary or secondary osteoporosis [1]. Primary osteoporosis is further divided into postmenopausal (type 1) and senile (type 2), while secondary osteoporosis results from other diseases, medications or external factors [1,2]. Women are generally at higher risk, particularly for postmenopausal osteoporosis, and the condition becomes more prevalent with age. Estrogen deficiency is the main driver of bone loss after menopause and is associated with apoptotic death of osteocytes in vivo [3]. As osteoporosis is often asymptomatic until fractures occur, global prevalence estimates vary widely, from 4.1% in the Netherlands to 52.0% in Turkey, with a meta-analysis indicating a prevalence of around 23.1% in women and 11.7% in men [2,4,5]. Treatments include pharmacological options such as oral and intravenous bisphosphonates (alendronate, ibandronate, risedronate, zoledronic acid), RANK-L inhibitors (denosumab), and anabolic agents (abaloparatide, romosozumab, teriparatide), as well as lifestyle interventions including nutrition, exercise, and calcium/vitamin D supplementation [6,7]. Despite available therapies, osteoporosis remains a growing global concern, highlighting the need for novel approaches.

Phytoestrogens are plant-derived compounds with structural similarities to estradiol, enabling estrogenic effects in vivo [8]. Major groups include isoflavones, coumestans, lignans, stilbenes, and prenylflavonoids [9,10,11]. Isoflavones are present in legumes (e.g., soy), cereals, fruits, and vegetables [12], coumestans in peas, sprouts, and beans, and lignans in grains, nuts, seeds, and beverages such as tea and wine [12,13]. Prenylflavonoids are highly lipophilic due to their flavonoid cores with farnesyl, prenyl, or geranyl side chains and exhibit antioxidant, anti-inflammatory, antimicrobial, antiparasitic, and estrogenic effects [14]. Sources of prenylflavonoids include hops, mulberry, figs, soybeans, and licorice [15].

Hops (*Humulus lupulus*) are primarily used in beer production [16]. The female hop inflorescence contains polyphenols, phenolic acids, proanthocyanidins, chalcones, and prenylflavonoids, with antibacterial and antifungal properties [17]. Historically, hops were used as sedatives, as well as for insomnia, digestive disorders, pain relief, and blood purification [18,19]. Modern studies indicate that hops alleviate postmenopausal symptoms, possess sedative properties, and influence osteoporosis [20,21,22,23,24]. Supercritical carbon dioxide extraction preserves bioactive prenylflavonoids, which can be used for dietary supplements [25,26,27]. The main hop prenylflavonoids are xanthohumol (XH), isoxanthohumol (IX), 6-prenylnaringenin (6PN), and 8-prenylnaringenin (8PN) [25]. XH and IXH exhibit minimal phytoestrogenic activity but serve as precursors for 8PN and 6PN via liver enzymes and gut microbiota [28]. 8PN is among the most potent phytoestrogens, binding estrogen receptors (ERs) with a preference for ERα in vivo, exerting osteoprotective effects by inhibiting bone resorption and increasing bone mineral density [29,30,31]. Although 8PN exhibits a broad range of beneficial effects, including metabolic, osteogenic, neuroprotective, and anti-inflammatory actions, there is an indication of potentially adverse endocrine and reproductive effects, particularly in males, highlighting the need for caution and further investigation of chronic and high-dose exposure before considering its therapeutic application [32].

Reactive oxygen species (ROS) can act as signaling molecules regulating bone cell differentiation and proliferation but, in excess, damage proteins, RNA, and DNA, inhibiting bone cell differentiation in bone tissue [33,34]. A meta-analysis reported a correlation between increased systemic oxidative stress and postmenopausal osteoporosis [35]; nevertheless, it is still not completely clear to what extent it represents a causal factor or a consequence of estrogen deficiency and aging, which is further complicated by other contributing factors, such as lifestyle and comorbidities [36,37,38]. In human studies, this distinction is difficult to resolve due to the lack of truly comparable healthy controls and the confounding effects of age-related changes. In this context, controlled animal models, including sham-operated groups, provide a useful approach to better understand these relationships.

Oxidative stress can influence bone remodeling, including osteoblast apoptosis and osteoclast differentiation, and compounds with antioxidant properties may modulate these effects in vitro and in animal models [35,39]. However, such effects may arise through multiple mechanisms and do not necessarily reflect direct free radical scavenging. Compounds commonly described as antioxidants can also act by modulating cellular signaling pathways involved in redox or inflammation regulation. For example, hop prenylflavonoids (primarily XH and 8PN) have shown antioxidant-related properties; however, they are relatively weak direct scavengers of ROS and are thought to act mainly through indirect mechanisms, including activation of the Keap1–Nrf2 pathway, modulation of glutathione metabolism, and regulation of inflammatory and metabolic signaling pathways (although higher doses may also elevate ROS, indicating dose-dependent and context-specific effects) [23]. Furthermore, while antioxidant compounds have demonstrated beneficial effects on bone-related parameters, evidence from clinical studies remains inconsistent, and there is still a need for large-scale clinical trials to demonstrate clear benefits [40,41].

Beyond the bone, estrogen deficiency is associated with significant metabolic and oxidative alterations in multiple tissues, including the liver and adipose tissue. This can lead to dyslipidemia, characterized by elevated serum triglycerides and cholesterol levels, and promotes the onset of cardiovascular diseases and metabolic syndrome [42]. Recent studies confirm that ovariectomy and/or menopause are associated with reduced hepatic fatty-acid oxidation, and increased lipogenesis and fat accumulation in liver, as well as increased risk of hepatic fibrosis, while estrogen signaling exerts protective effects on the liver [43,44,45,46]. Also, different phytoestrogens have been shown to mitigate the metabolic disorders and consequences of NAFLD in postmenopausal women [47].

Currently, there is limited data on combined plant-derived estrogenic compounds and bisphosphonates. This study is part of a larger project designed to investigate the early effects of alendronate, hop extract, and their combination on bone-related outcomes in ovariectomized rats. The primary endpoints of that study, including bone microarchitecture, are reported separately [48]. These findings confirmed successful induction of the ovariectomy model in this experimental cohort and report on the effect of the studied compounds and their combinations in the model. The current manuscript focuses on short-term exploratory outcomes related to systemic metabolism, oxidative stress, and tissue-specific responses in liver and adipose tissue, for which OV-related baseline differences may be less pronounced. To investigate these effects, this study employs lipidomic, biochemical, and histological analyses in ovariectomized rats. Combining matrix-assisted laser desorption/ionization time-of-flight mass spectrometry (MALDI-TOF MS) lipidomics with morphometry and biochemical assays provides insight into tissue-specific metabolic and oxidative stress responses following combined short-term alendronate and hop extract therapy. Given the insufficient and heterogeneous background knowledge on the early metabolic and oxidative effects of ovariectomy and possible modulation by the interventions used, this study was designed as exploratory. Therefore, no primary outcome was defined, and multiple biological endpoints were measured to assess the possible treatment-related effects.

## 2. Materials and Methods

### 2.1. Animals and Experimental Design

The study is fully compliant with Croatian legislation on animal welfare and protection and the Guide for the Care and Use of Laboratory Animals, DHHS (NIH) Publ # 86-23 [49]. All procedures performed on animals were approved by: the Committee for bioethics and animal welfare of the Faculty of Science, University of Zagreb (Class: 643-02/19-01/3; Reg. No.: 251-58-10617-19-704; date of approval: 17 October 2019), the Croatian National Ethical Committee for animals used for scientific research (EP 233/2020, Class: UP/I-332-01/19-01/115; Reg. No.: 525-10/1315-20-6; date of approval: 27 March 2020), and the Ethical Committee of the Faculty of Medicine Osijek (Class: 602-04/22-08/02; Reg. No.: 2158-61-46-22-16; date of approval: 25 February 2022).

Seventy female Wistar rats, six months old and weighing 206–280 g, were used in this study. Animals were housed in standard laboratory conditions with a 12-h light/dark cycle at 24 °C and provided with ad libitum access to standard chow (4 RF 21, Mucedola S.R.L., Milan, Italy) and water. Rats were randomly assigned into seven groups (*n* = 10 per group). Sample size was based on prior studies and ethical considerations. One group underwent a sham operation and served as the healthy control (C), while the remaining six groups underwent bilateral ovariectomy to induce estrogen-deficiency-associated osteoporosis. Surgical procedures were performed under inhalatory anesthesia using sevoflurane (Sevorane, Abbott Laboratories, Chicago, IL, USA). Postoperative analgesia was provided as needed with ketoprofen (2–5 mg/kg, intraperitoneally). Animals were maintained for one-month post-surgery to allow for the development of osteoporotic changes.

After this period, the ovariectomized animals were assigned to six treatment groups treated over the next two weeks. The ovariectomized untreated (OV) group received 1 mL intragastric (i.g.) propylene glycol as vehicle. The low-dose alendronate (AL) group received 1 mg/kg i.g. alendronate daily, and the high-dose alendronate (AH) group received 2 mg/kg daily [50,51]. The low-dose alendronate plus hop extract (AL-X) group received 1 mg/kg alendronate every other day with 60 mg/kg hop extract administered on intervening days, while the high-dose combination (AH-X) group received 2 mg/kg alendronate every other day with 60 mg/kg hop extract on alternating days. The last group (X) received only hop extract at 60 mg/kg i.g. daily [52]. The control group also received 1 mL i.g. propylene glycol daily. Animals were group-housed in standard cages (430 × 290 × 201 mm), with 5 animals per cage. Each experimental group was distributed across 2 cages, with all animals within a cage receiving the same treatment. Detailed identification of individual cage allocation within treatment groups was not possible, as these records were not preserved from the original experiment. During the post-ovariectomy recovery period, two animals died (one in C group, and one in X group, resulting in a final sample size of *n* = 68. The deaths were not considered related to treatment.

During each administration, animals were briefly anesthetized with sevoflurane to minimize stress. Doses and schedules were based on previous studies optimizing oral alendronate and hop extract administration in ovariectomized rats. At the end of the treatment period, animals were euthanized under deep anesthesia using intraperitoneal ketamine (75 mg/kg, Narketan^®^, Bioveta, Ivanovice na Hané, Czech Republic) and xylazine (10 mg/kg, Xylapan^®^, Bioveta, Ivanovice na Hané, Czech Republic). Blood was collected at euthanasia in non-heparinized tubes, allowed to clot, and centrifuged at 3000× *g* for 10 min to obtain serum, which was stored at −80 °C until analysis. Liver, visceral adipose tissue (VAT), and kidneys were collected for subsequent analyses and either fixed in 10% neutral-buffered formalin (NBF) and embedded in paraffin blocks or stored at −20 °C until further processing. The experimental design and animal procedures were originally developed to address primary skeletal outcomes, with additional analyses performed on collected samples as outlined above.

### 2.2. Compounds and Preparation

Alendronate was administered as Aledox 70 tablets (Belupo, Koprivnica, Croatia), and hop extract was supplied as XanthoFlav™ (Hopsteiner, New York, NY, USA). All compounds were freshly suspended in propylene glycol prior to administration to achieve the desired intragastric doses. High-performance liquid chromatography (HPLC) analysis of XanthoFlav™ indicated a prenylflavonoid composition of 75% xanthohumol, 1.7% 6-prenylnaringenin, 0.4% isoxanthohumol, and 0.3% 8-prenylnaringenin.

### 2.3. Analysis of Serum Total Cholesterol and Triglycerides

Serum total cholesterol and triglyceride concentrations were determined using commercially available colorimetric enzymatic assays (Cholesterol liquicolor and Triglycerides liquicolor mono, HUMAN, Wiesbaden, Germany). Standard curves were prepared using known concentrations of cholesterol (5.17 mmol/L) and triglycerides (2.28 mmol/L). Serum samples (2 µL) were incubated with assay reagents in 96-well plates at room temperature for 10 min, and absorbance was measured at 490 nm. Concentrations were calculated from the standard curves and expressed in mmol/L.

### 2.4. Analysis of Oxidative Stress and Liver Biochemistry

#### 2.4.1. Serum Nitric Oxide and Arginase Activity

Serum nitric oxide (NO) levels were assessed by measuring nitrites (NO_2_^−^) using a Griess reagent-based assay (Promega, Madison, WI, USA). Using the supplied nitrite standard, serial dilutions were made, and calibration curves were obtained as per manufacturer’s instructions. Serum samples (50 µL) and nitrite standards were prepared in triplicate in 96-well plates, incubated with sulfanilamide for 10 min, followed by N-1-naphthylethylenediamine dihydrochloride for 10 min, and absorbance measured at 515 nm using iMark™ Microplate Reader (Bio-Rad Laboratories, Hercules, CA, USA). Nitrite concentrations (µmol/L) were determined from the average absorbance of technical triplicates and the calibration curve.

Arginase activity in serum was measured using a commercial kit (Sigma-Aldrich, St. Louis, MO, USA). Urea standards were prepared as per manufacturer’s instructions, and serum samples were diluted (1:10 in water) and pipetted into 96-well plates in duplicate (control and experimental). Arginase substrate reagent was added in experimental wells and incubated at 37 °C for 2 h. After that, a color-developing reagent that reacts with urea was added to all wells, while the control set received the arginase substrate and was incubated for 1 h at room temperature. The result was quantified colorimetrically at 415 nm using iMark™ Microplate Reader (Bio-Rad Laboratories, Hercules, CA, USA). Enzyme activity was expressed in units per liter, where one unit catalyzes the conversion of 1 µmol L-arginine to ornithine and urea per minute at pH 9.5 and 37 °C.

#### 2.4.2. Total Protein Determination

To normalize malondialdehyde and other biochemical measurements, total protein content in liver and kidney homogenates was quantified using the Bradford assay [53]. The Bradford reagent was prepared by dissolving 50 mg of Coomassie Brilliant Blue G-250 (Carl Roth, Karlsruhe, Germany) in 50 mL methanol (Carlo Erba, Milan, Italy), then adding 100 mL of 85% phosphoric acid (Sigma, St. Louis, MO, USA) and diluting with distilled water to a final volume of 1 L. The reagent was filtered through a 0.2 µm PES filter (Nalgene™, Thermo Fisher Scientific, Waltham, MA, USA) and stored at 4 °C. A calibration curve was generated using serial dilutions of bovine serum albumin (Sigma, St. Louis, MO, USA) in PBS, ranging from 0.125 to 20 mg/mL. Aliquots of 1 µL of homogenate or standard were pipetted in technical triplicate into 96-well plates, and 250 µL of Bradford reagent was added to each well. After 10 min of incubation at room temperature, absorbance was measured at 595 nm using iMark™ microplate reader. Protein concentrations were calculated from the calibration curve and average absorbance of the technical triplicate and expressed as mg/mL.

#### 2.4.3. Lipid Peroxidation in Liver and Kidney Tissue

Malondialdehyde (MDA) levels were measured in liver and kidney homogenates as an index of lipid peroxidation. For each sample, 100 µL of homogenate was transferred into a 2 mL plastic tube and 1.6 mL of freshly prepared thiobarbituric acid (TBA; Sigma, St. Louis, MO, USA) reagent. The reagent was prepared by mixing 10 mL of 8.1% sodium dodecyl sulfate (SDS; Sigma, St. Louis, MO, USA) in distilled water with 75 mL of 20% acetic acid containing 0.85% HCl (pH 3.5) and 75 mL of 0.8% thiobarbituric acid. Samples were incubated for 1 h in a water bath at 95 °C, then cooled on ice to room temperature and centrifuged for 12 min at 5000× *g* and 4 °C (Sigma 1 15PK, Sigma Laborzentrifugen GmbH, Osterode am Harz, Germany) to remove insoluble components. The absorbance of the resulting supernatant was measured at 523 nm using 1 mL polystyrene cuvettes with a 1 cm path length in a Lambda 25 UV/vis spectrophotometer (PerkinElmer, Waltham, MA, USA). MDA concentrations were calculated using an extinction coefficient of ε = 1.56 × 10^5^ M^−1^ cm^−1^ and normalized to total protein content, as measured by the Bradford assay. Results were expressed as nmol MDA per mg protein.

#### 2.4.4. Protein Carbonyls

Protein carbonyls, indicative of oxidative protein damage, were measured in liver and kidney homogenates using 2,4-dinitrophenylhydrazine (DNPH; Merck, Darmstadt, Germany) according to a modified Levine method [54]. For each sample, 10 µL of homogenate was diluted 1:4 with PBS and prepared in duplicate, with one set serving as a blank. Proteins were precipitated by adding 100 µL of 30% trichloroacetic acid (Merck, Darmstadt, Germany), mixed, and centrifuged at 5000× *g* for 10 min at room temperature (Sigma 1-15PK). The pellet was solubilized in 100 µL of 1:1 HCl:acetone to remove hemoglobin and centrifuged at 15,000× *g* for 10 min at 4 °C. For carbonyl derivatization, 250 µL of 10 mM DNPH in 2 N HCl was added to the test samples, and 250 µL 2 N HCl to the blanks. Samples were incubated for 1 h in the dark with mixing every 15 min, then centrifuged at 15,000× *g* for 10 min at 4 °C. Excess DNPH was removed by two washes with 250 µL ethanol:ethyl acetate (1:1) with centrifugation at 15,000× *g* for 10 min at 4 °C. The final pellet was solubilized in 125 µL of 6 M guanidine hydrochloride. Absorbance was measured in quartz cuvettes at 375 nm for DNPH-treated samples and 280 nm for blanks (Lambda 25 UV/vis), to allow for normalization to protein content. Carbonyl content was calculated using an extinction coefficient of 22 × 10^3^ M^−1^ cm^−1^ and expressed as µmol carbonyl per mg protein. Calibration curves were constructed using bovine serum albumin dissolved in guanidine hydrochloride.

#### 2.4.5. Analysis of Catalase Activity

Catalase activity in liver and kidney homogenates was determined according to Sinha, using previously measured total protein for normalization [55]. Serial dilutions of hydrogen peroxide (6.25 mM to 200 mM) were prepared, and 330 µL of each dilution was mixed in triplicate with 660 µL of 1.25% potassium dichromate in 75% acetic acid. Samples were heated at 100 °C for 10 min and then cooled on ice. For calibration, 200 µL of each hydrogen peroxide standard dilution was pipetted into 96-well plates, and absorbance was measured at 595 nm using an iMark™ Microplate Reader, and a calibration curve was prepared. For measurement, homogenates were diluted 1:50 in PBS; 66 µL of diluted sample was mixed with 264 µL of 0.2 M hydrogen peroxide to achieve 160 mM hydrogen peroxide in the sample. After 1 min, 660 µL of 1.25% potassium dichromate in 75% acetic acid was added, and samples were heated at 100 °C for 10 min to react with the residual hydrogen peroxide, forming a colored chromic acetate complex. Two hundred microliters of each reaction mixture were transferred to 96-well plates, and absorbance was measured at 595 nm using the same reader. Catalase activity was calculated from the hydrogen peroxide standard curve and expressed as millimoles of hydrogen peroxide decomposed per second per microgram of total protein.

#### 2.4.6. Liver Triglycerides and Cholesterol

Hepatic concentrations of total cholesterol and triglycerides were measured using commercial colorimetric enzymatic assays (Cholesterol liquicolor and Tryglicerides liquicolor mono, HUMAN, Wiesbaden, Germany) on previously prepared liver homogenates and using the same protocol as for serum triglycerides and cholesterol (see above). The results were normalized to previously obtained total protein content and expressed as µmol per gram of protein.

### 2.5. Histology and Morphometric Analysis of Liver and Perigonadal Adipose Tissue

#### 2.5.1. Liver Tissue

The central portion of liver lobe fixed in 10% NBF was paraffin-embedded and sectioned at 6 µm. Sections were mounted on glass slides and stained for collagen fibers with 0.1% Sirius Red F3B in saturated aqueous picric acid (Puchtler method). After deparaffinization and rehydration, slides were incubated in Puchtler’s solution for 1 h at room temperature, rinsed twice in 0.5% aqueous acetic acid, dehydrated, cleared in xylene, and mounted with Canada balsam. Whole-slide images were acquired with a Zeiss Axiovert 200M microscope and Axiocam MRc camera (Carl Zeiss, Oberkochen, Germany) using the “MosaiX” tiling function under a 20× objective in Zeiss AxioVision software. Tiled images were stitched into a single composite in FIJI v2.10.0 using the Grid/Collection stitching plugin v3.1.9 [56,57]. Background was removed using GIMP v2.10.34 (GNU image manipulation program, GIMP Development Team, https://www.gimp.org). Collagen masks were generated via color thresholding in FIJI, and collagen area was measured relative to total tissue area. Values were converted from pixels to µm^2^, expressed as a percentage of total tissue area, and exported for statistical analysis.

#### 2.5.2. Analysis of Perigonadal Adipose Tissue

Perigonadal adipose tissue samples were fixed in 10% NBF, paraffin-embedded, and sectioned at 6 µm using a rotary microtome Slee CUT 4060 (Slee, Mainz, Germany). Sections were stained for collagen using the same picrosirius staining protocol as adipose tissue. Whole-slide images were acquired with the abovementioned setup and software under a 10× objective. Tiled images were stitched into a single composite in FIJI using the Grid/Collection stitching plugin. Adipocyte morphometry was performed with the Adiposoft plugin in FIJI, automatically excluding damaged cells [58]. Adipocyte diameters were restricted to 40–150 pixels. The analyzed parameters were mean adipocyte area (µm^2^), equivalent diameter (µm), and cell number.

### 2.6. MALDI-TOF MS Analysis

Matrices for MALDI-TOF MS analysis were prepared at a concentration of 10 mg/mL. 2,5-Dihydroxybenzoic acid (DHB; Sigma-Aldrich, St. Louis, MO, USA) was dissolved in high-purity methanol, while 9-aminoacridine (9-AA; Sigma-Aldrich, St. Louis, MO, USA) was dissolved in a 40:60 mixture of high-purity acetonitrile and isopropanol. Both matrices were stored at 4 °C until use.

Liver lipids were isolated from homogenized tissue using a high-purity chloroform:isopropanol method to remove hemoglobin and pigments [59]. Total lipid isolates were dried under a nitrogen stream at 45 °C and stored at −80 °C until analysis.

Polar and nonpolar lipids from perigonadal adipose tissue were extracted using a modified Bligh and Dyer method [60]. Polar and nonpolar phases were collected separately, dried under nitrogen at 45 °C, and stored at −80 °C until analysis.

Prior to MALDI-TOF MS analysis, each lipid sample was resuspended in 100 µL of high-purity chloroform:methanol (3:7). 10 µL of sample were mixed with 10 µL of the appropriate matrix (DHB for positive mode, 9-AA for negative mode), and 2 µL of the mixture was applied onto a MALDI target metal plate (Cat. No. 8280781, Bruker, Billerica, MA, USA). Red phosphorus was applied for external calibration.

Data acquisition was performed on a Bruker UltrafleXtreme MALDI-TOF/TOF MS system (Bruker, Billerica, MA, USA) equipped with a Smartbeam laser over a mass range of 200–2000 Da in both positive and negative reflectron modes. Spectra were acquired using a 355 nm Nd:YAG laser at 90% intensity and 2000 Hz with 10 shots per sample and an accumulation of 15,000 shots per spectrum. Ion source voltage was 20 kV (source 1), 17.75 kV (source 2), lense voltage 8.3 kV with detector gain 20× in negative mode and 13.6× in positive mode with digitizer 5.0 GS/s. Data were processed in Bruker FlexAnalysis software v3.4 using the Snap peak detection algorithm, Savitzky–Golay smoothing, and TopHat baseline correction. Subsequent statistical and KEGG pathway analyses were conducted in R using the packages: matrixTests, roperators, FELLA, KEGGREST, igraph and magrittr [61,62,63,64,65,66,67]. Raw spectra were binned across the *m*/*z* range of 200–2000 using a bin size of 0.1 Da. Bins with a summed signal intensity below 1% of the maximum observed signal were excluded prior to further analysis.

### 2.7. Statistical Analysis

For statistical analyses, the individual animal was considered the experimental unit. Data preparation and tabulation were performed in Microsoft Excel, statistical analysis (except MALDI-TOF MS data) was done using software Past v4.09 [68]. Outliers within groups were identified and removed using Grubbs’ and Dixon’s Q tests. Normality of data distribution was assessed using the Shapiro–Wilk test, and homogeneity of variance was tested with Levene’s test. For normally distributed data with homogeneous variance, comparisons among groups were performed using one-way analysis of variance (ANOVA), with *p*-values reported. Non-normally distributed data were analyzed using the Kruskal–Wallis test, with χ^2^ and *p*-values reported. Post hoc comparisons were performed using Tukey–Kramer tests for normally distributed data and Dunn’s test with Bonferroni correction for non-normal data. To account for the factorial structure of the experimental design, outcomes were additionally analyzed using two-way ANOVA to assess the main effects of alendronate and hop extract and their interaction, as well as to better interpret possible treatment-specific and interaction effects. For MALDI-TOF MS data, comparisons between experimental and control groups were conducted using Welch’s *t*-test in R with Benjamini–Hochberg false discovery rate used for *p*-value adjustment. All *p*-values are two-tailed, with significance set at α = 0.05. For consistency and comparability across datasets, all graphical data are presented as box-and-whisker plots using median and interquartile range. This study was exploratory in design, no primary outcome was specified, and multiple outcomes were analyzed. Except for MALDI-TOF MS data, no formal correction for multiple comparisons was applied; therefore, the findings are presented as exploratory and should be interpreted accordingly.

## 3. Results

### 3.1. Serum Total Cholesterol and Triglycerides

**Figure 1:**
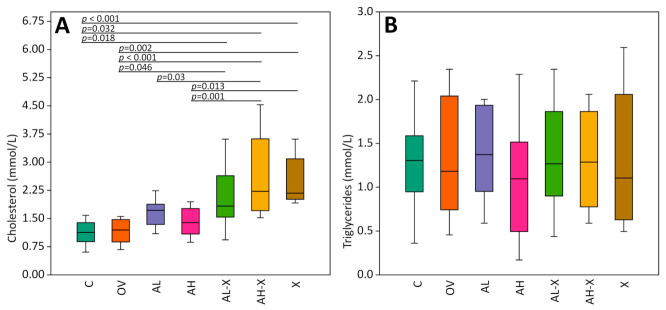
Serum cholesterol (**A**) and triglyceride (**B**) concentrations. Six-month-old rats were ovariectomized (*n* = 60) or sham-operated (*n* = 10). One month later, animals were assigned to treatment groups for two weeks and then euthanized (*n* = 9–10 animals per group). Differences were tested using one-way ANOVA with Tukey–Kramer post hoc test, and significant differences are labeled in the figure. Horizontal line: median; box: interquartile range; vertical lines with delimiter: minimum and maximum. C—sham-operated group; OV—untreated ovariectomized group; AL—ovariectomized low-alendronate group; AH—ovariectomized high-alendronate group; AL-X—ovariectomized low-alendronate and hop extract group; AH-X—ovariectomized high-alendronate and hop extract group; X—ovariectomized animals, hop extract group.

Ovariectomy did not affect serum total cholesterol levels (Figure 1A). Two-way ANOVA revealed a significant main effect of hop extract on serum total cholesterol (*p* < 0.001), while neither alendronate nor the interaction between the two showed a significant effect (Table 1). Hop extract increased cholesterol levels regardless of alendronate treatment. Consistent with this, pairwise comparisons shown in Figure 1A demonstrate that all groups receiving hop extract had significantly increased serum cholesterol concentrations compared to the C and OV groups, with the AH-X group exhibiting the highest values.

In contrast, serum triglyceride levels were not significantly affected by the experimental interventions (*p* > 0.05; Figure 1B).

### 3.2. Oxidative Stress and Liver Biochemistry

#### 3.2.1. Serum Nitrites and Arginase Activity

**Figure 2:**
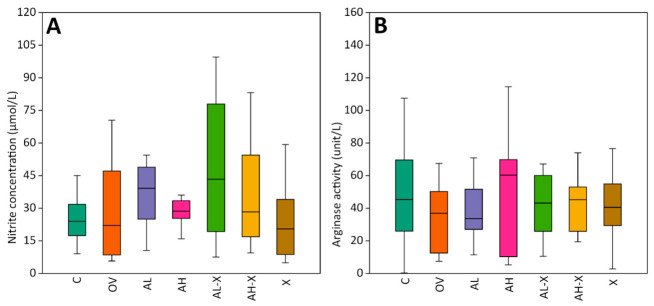
Results of the analysis of serum nitrite concentration (**A**) and arginase activity (**B**). Six-month-old rats were ovariectomized (*n* = 60) or sham-operated (*n* = 10). One month later, animals were assigned to treatment groups for two weeks and then euthanized (*n* = 9–10 animals per group). Differences between groups were tested by one-way ANOVA analysis with Tukey–Kramer post hoc test. No statistically significant differences between groups were recorded. Horizontal line: median; box: interquartile range; vertical lines with delimiters: minimum and maximum. C—sham-operated group, OV—untreated ovariectomized group, AL—ovariectomized low alendronate group, AH—ovariectomized high alendronate group, AL-X—ovariectomized low alendronate and hop extract group, AH-X—ovariectomized high alendronate and hop extract group, X—ovariectomized animals, hop extract group.

Serum nitric oxide concentration was measured through nitrite concentrations (µmol/L), and serum arginase activity was expressed as arginase units per liter. Serum nitrite concentrations and arginase activity were not affected by ovariectomy. No significant effects of alendronate, hop extract, or their combination on these parameters were found (all *p* > 0.05, Figure 2A,B).

#### 3.2.2. Lipid Peroxidation in Liver and Kidney

**Figure 3:**
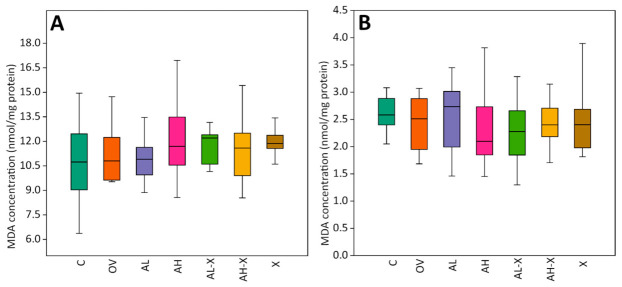
Results of analysis of lipid peroxidation by measuring the concentration of malondialdehyde (MDA) in liver (**A**) and kidney (**B**) tissue. Six-month-old rats were ovariectomized (*n* = 60) or sham-operated (*n* = 10). One month later, animals were assigned to treatment groups for two weeks and then euthanized (*n* = 9–10 animals per group). Differences between groups were tested by one-way ANOVA analysis with Tukey–Kramer post hoc test. No statistically significant differences were recorded between groups. Horizontal line: median; box: interquartile range; vertical lines with delimiter: minimum and maximum. C—sham-operated group; OV—untreated ovariectomized group; AL—ovariectomized low-alendronate group; AH—ovariectomized high-alendronate group; AL-X—ovariectomized low-alendronate and hop extract group; AH-X—ovariectomized high-alendronate and hop extract group; X—ovariectomized animals, hop extract group.

Lipid peroxidation was analyzed via MDA concentrations in liver and kidney tissue, expressed as nmol/L and normalized to total protein. One-way ANOVA analysis revealed no significant differences in MDA concentrations for liver (*p* = 0.56) and kidney (*p* = 0.75) tissue (Figure 3A and Figure 3B, respectively).

### 3.3. Protein Carbonyl

**Figure 4:**
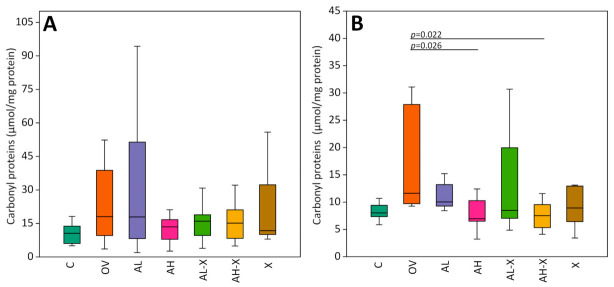
Results of analysis of protein carbonyl content in liver (**A**) and kidney (**B**) tissue. Six-month-old rats were ovariectomized (*n* = 60) or sham-operated (*n* = 10). One month later, animals were assigned to treatment groups for two weeks and then euthanized (*n* = 9–10 animals per group). Differences between groups were tested using the Kruskal–Wallis test with Dunn’s post hoc test and Bonferroni’s *p* value correction, and significant differences between groups and significant differences are labeled in the figure. Horizontal line: median; box: interquartile range; vertical lines with delimiters: minimum and maximum. C—sham-operated group; OV—untreated ovariectomized group; AL—ovariectomized low-alendronate group; AH—ovariectomized high-alendronate group; AL-X—ovariectomized low-alendronate and hop extract group; AH-X—ovariectomized high-alendronate and hop extract group; X—ovariectomized animals, hop extract group.

In liver samples, no significant differences in the protein carbonyl content were recorded between the examined groups (Figure 4A).

In kidney tissue, two-way ANOVA revealed a significant main effect of alendronate (*p* = 0.011) and a significant interaction between alendronate and hop extract (*p* = 0.042), while no main effect of hop extract alone was observed (Table 1). Pairwise comparisons (Figure 4B) indicated decreased carbonyl content in the groups receiving high-dose alendronate compared to OV group.

### 3.4. Catalase Activity

**Figure 5:**
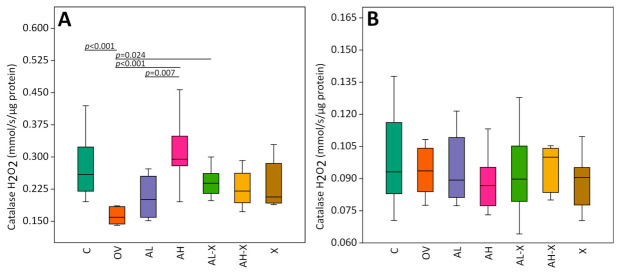
Catalase activity in liver tissue (**A**) and kidney tissue (**B**) measured by the rate of hydrogen peroxide decomposition and normalized to total tissue proteins. Six-month-old rats were ovariectomized (*n* = 60) or sham-operated (*n* = 10). One month later, animals were assigned to treatment groups for two weeks and then euthanized (*n* = 9–10 animals per group). Differences between groups were tested by Kruskal–Wallis test with Dunn’s post hoc test and Bonferroni’s *p* value correction, and significant differences are labeled in the figure. Horizontal line: median; box: interquartile range; vertical lines with delimiter: minimum and maximum. C—sham-operated group; OV—untreated ovariectomized group; AL—ovariectomized low-alendronate group; AH—ovariectomized high-alendronate group; AL-X—ovariectomized low-alendronate and hop extract group, AH-X—ovariectomized high-alendronate and hop extract group; X—ovariectomized animals, hop extract group.

Catalase activity (the rate of hydrogen peroxide decomposition and normalized to total tissue proteins) was compared for liver and kidney. A significant main effect of alendronate (*p* < 0.001) and a significant interaction between alendronate and hop extract (*p* < 0.001) was recorded on catalase activity in liver tissue, whereas hop extract alone had no significant effect (Table 1). Kruskal–Wallis test showed significant difference for liver as well (χ^2^ = 32.49, *p* = 0.013); differences between groups are shown in Figure 5A. Ovariectomy significantly decreased catalase activity in liver. Significantly higher activity was recorded in the AH and AL-X groups compared to OV, and significantly lower activity in the AL group compared to the AH group. Catalase activity in kidney tissue (Figure 5B) did not differ significantly between the groups.

### 3.5. Liver Total Cholesterol and Triglycerides

**Figure 6:**
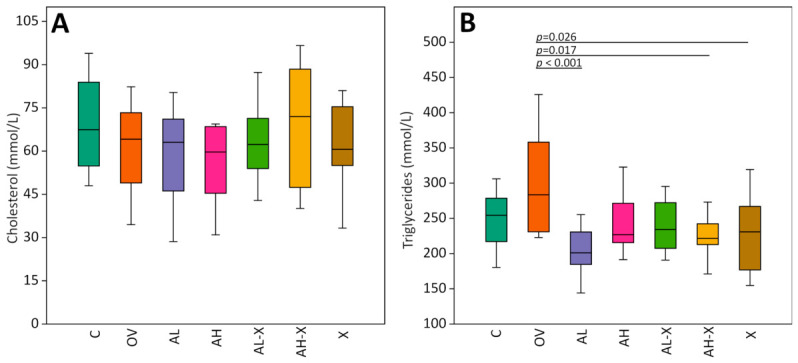
Total cholesterol (**A**) and triglyceride concentrations (**B**) in liver tissue. Six-month-old rats were ovariectomized (*n* = 60) or sham-operated (*n* = 10). One month later, animals were assigned to treatment groups for two weeks and then euthanized (*n* = 9–10 animals per group). Differences between groups were tested by one-way ANOVA analysis with Tukey–Kramer post hoc test, and significant differences are labeled in the figure. Horizontal line: median; box: interquartile range; vertical lines with delimiter: minimum and maximum. C—sham-operated group; OV—untreated ovariectomized group; AL—ovariectomized low-alendronate group; AH—ovariectomized high-alendronate group; AL-X—ovariectomized low-alendronate and hop extract group; AH-X—ovariectomized high-alendronate and hop extract group; X—ovariectomized animals, hop extract group.

Total cholesterol concentration in liver tissue did not differ significantly between groups (one-way ANOVA, *p* = 0.56). For liver triglycerides, two-way ANOVA showed a significant main effect of alendronate (*p* = 0.030) and a significant interaction between alendronate and hop extract (*p* = 0.004), while hop extract alone had no significant effect (Table 1). One-way ANOVA (*p* = 0.035) showed that triglyceride concentrations were significantly higher in the OV group compared to the AL, AH-X and X groups. Although the OV group had the highest liver triglyceride levels, they did not differ significantly from the C (Figure 6).

### 3.6. Results of Histology and Morphometric Analysis of Liver and Perigonadal Adipose Tissue

#### 3.6.1. Collagen Proportion in Liver Tissue

**Figure 7:**
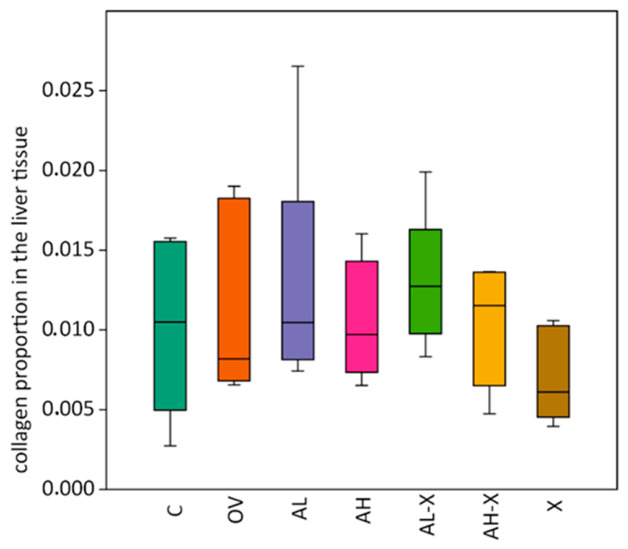
Results of the analysis of collagen content in liver tissue. Six-month-old rats were ovariectomized (*n* = 60) or sham-operated (*n* = 10). One month later, animals were assigned to treatment groups for two weeks and then euthanized (*n* = 9–10 animals per group). Differences between groups were tested by ANOVA analysis with Tukey–Kramer post hoc test. No significant differences were found between groups. Horizontal line: median; box: interquartile range; vertical lines with delimiters: minimum and maximum. C—sham-operated group; OV—untreated ovariectomized group; AL—ovariectomized low-alendronate group; AH—ovariectomized high-alendronate group; AL-X—ovariectomized low-alendronate and hop extract group; AH-X—ovariectomized high-alendronate and hop extract group; X—ovariectomized animals, hop extract group.

**Figure 8:**
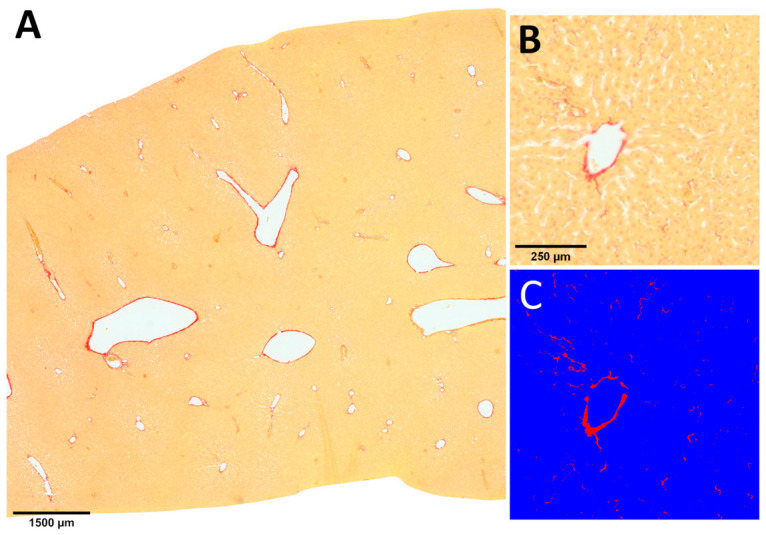
An example of a histological liver section prepared for collagen content analysis. The image shows the entire surface of the analyzed section (**A**) enlarged detail of the section (**B**) and the same detail processed in GIMP software to create the mask used for analysis (**C**). In the mask, collagen is highlighted in red, and the rest of the tissue in blue. Picrosirius red staining, 20× objective, scale bars: 1500 µm (**A**) and 250 µm (**B**).

The area of photographed tissue and collagen was measured on sections of liver tissue. The values obtained were expressed as the proportion of collagen in the total area of liver tissue. The proportion of collagen in liver tissue did not differ significantly between groups (one-way ANOVA, *p* = 0.51, Figure 7). Figure 8 shows an example of analyzed liver section and a mask prepared using GIMP software v2.10.34 for subsequent analysis.

#### 3.6.2. Perigonadal Adipose Tissue Morphometry

**Figure 9:**
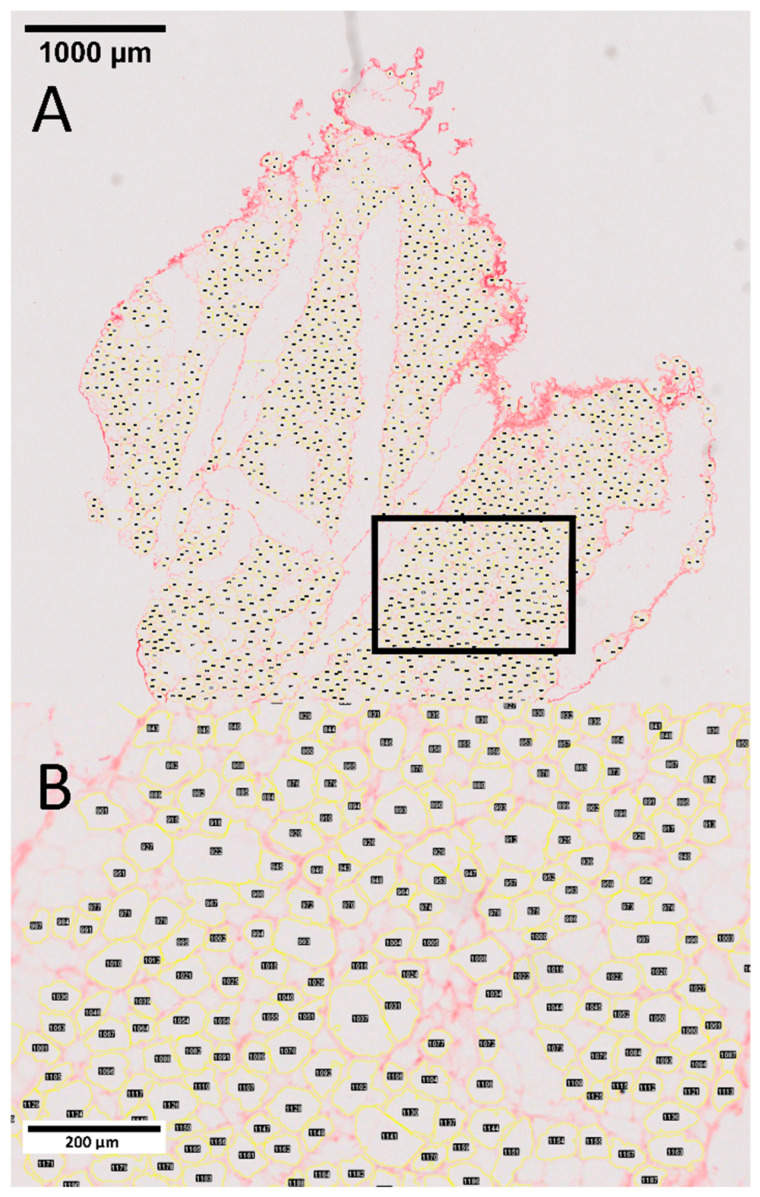
An example of marked adipocytes in perigonadal adipose tissue after analysis with the Adiposoft add-on in FIJI software v2.10.0. The image shows the entire stitched section of perigonadal adipose tissue (**A**) and a magnified detail from this image (indicated by a black rectangle) with labeled adipocytes (**B**). Each recognized adipocyte is marked and numbered in small black rectangles, and yellow lines delineate adipocyte borders. Staining: picrosirius red; objective: 10×; scale: 1000 µm (**A**), 200 µm (**B**).

On perigonadal adipose tissue sections stained with picrosirius, adipocyte morphology was clearly visible and identified well using Adiposoft plugin v1.16 for FIJI (Figure 9). Adipocyte areas (µm^2^), adipocyte number, and adipocyte equivalent diameter (µm) were measured. The adipocyte number per tissue area (/mm^2^) was also calculated.

**Figure 10:**
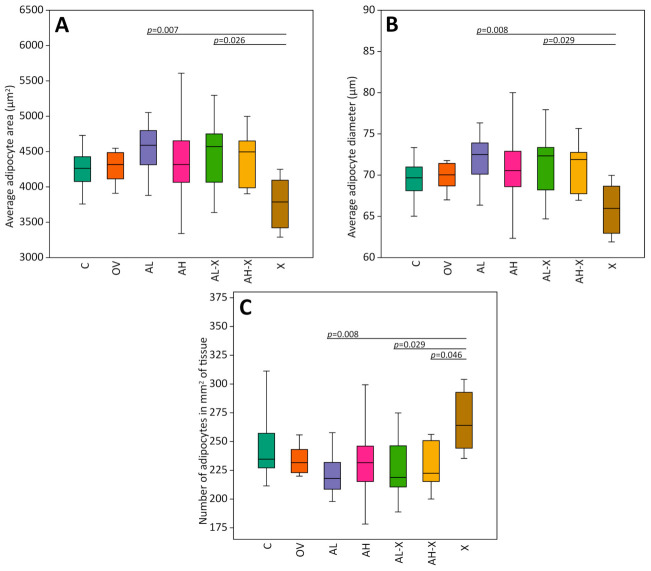
Results of perigonadal adipose tissue analysis. The figure shows the average adipocyte area (**A**), the average adipocyte diameter (expressed as equivalent diameter) (**B**) and the number of adipocytes per tissue area (**C**). Six-month-old rats were ovariectomized (*n* = 60) or sham-operated (*n* = 10). One month later, animals were assigned to treatment groups for two weeks and then euthanized (*n* = 9–10 animals per group). Differences between groups were tested by one-way ANOVA analysis with Tukey–Kramer post hoc test, and significant differences are labeled in the figure. Horizontal line: median; box: interquartile range; vertical lines with delimiters: minimum and maximum. C—sham-operated group; OV—untreated ovariectomized group; AL—ovariectomized low-alendronate group; AH—ovariectomized high-alendronate group; AL-X—ovariectomized low-alendronate and hop extract group AH-X—ovariectomized high-alendronate and hop extract group,; X—ovariectomized animals, hop extract group.

Two-way ANOVA showed a significant main effect of alendronate on average adipocyte area (*p* = 0.008), diameter (*p* = 0.007), and adipocyte number per tissue area (*p* = 0.007), while no significant effects of hop extract or interaction were observed (Table 1). Pairwise comparisons showed significant differences in the same parameters (*p* = 0.016, 0.016, and 0.013, respectively). Average adipocyte area and average adipocyte diameter were significantly lower in the X group compared to the AL group and AL-X group, and the number of adipocytes per square mm was significantly higher in the X group compared to the AL, AL-X and AH-X groups. However, the X group did not differ significantly from OV, nor did OV differ from C (Figure 10).

### 3.7. Results of MALDI-TOF MS Analysis

#### 3.7.1. Liver

Significant differences in tentatively annotated hepatic metabolites based on adjusted *p*-values are summarized in Table 2. They are grouped by metabolite class (amino acid metabolites including nucleotide derivatives; fatty acids and lipid mediators; glycerophospholipids, glycosphingolipids, and sphingolipids; oxidative stress markers, and steroids and derivatives).

When compared to the control group (C), L-histidine was increased in the AL-X group. Deoxyuridine monophosphate (dUMP) was reduced in the AH group and increased in the X group. L-Argininosuccinate was decreased in the AH and AL-X groups, while an increase was observed in the X group. Decanoic acid was markedly reduced in AH and AL-X and increased in X group, palmitic acid was also increased in the X group, and stearic acid in the AH group. 2,3-Bis(O-phytanyl)-sn-glycerol-1-phosphate was decreased in the AH group and increased in the X group. Ophthalmate was reduced in AH and AL-X and increased in the X group. Thyroxine (T4) was elevated in X compared to the C group.

When compared to the OV group, fewer significant differences were observed. Decanoic acid was reduced in the AH and X groups, stearic acid was increased in the AH group, and palmitic acid was decreased in the X group. 2,3-Bis(O-phytanyl)-sn-glycerol-1-phosphate was reduced in the X group.

Overall, the highest number of significant alterations relative to the C group was observed in the AH group, whereas the X group showed the most significant differences overall. Fewer differences were detected when comparisons were performed against the OV group.

#### 3.7.2. Perigonadal Adipose Tissue

Significant alterations in perigonadal adipose tissue metabolites based on adjusted *p*-values are summarized in Table 3. They are grouped by metabolite class (fatty acids, fatty acyl derivatives and lipid mediators; glycerophospholipids, glycosphingolipids, and sphingolipids; steroids and derivatives).

In comparison to the control group (C), L-palmitoylcarnitine was increased in the AH group, as well as sphinganine-1-phosphate and estrone-3-glucuronide. In contrast, arachidic acid (C20:0) and 3α,7α-dihydroxy-5β-cholestane were reduced in AH.

Within the X group (vs. C), multiple long-chain fatty acids were elevated, including arachidic acid (C20:0), eicosenoic acid (C20:1), erucic acid (C22:1), behenic acid (C22:0), nervonic acid (C24:1), and adrenic acid (C22:4).

When compared to the OV group, L-palmitoylcarnitine was increased in the AH group. Eicosenoic acid (C20:1) was reduced in X relative to OV. No other metabolites reached statistical significance after adjustment for multiple testing.

Overall, the most pronounced differences relative to C were observed in the X group, primarily involving long-chain fatty acids, whereas fewer changes were detected when comparisons were performed against OV. In fact, all of the significant changes fell into either the AH or X group.

## 4. Discussion

This study evaluated the short-term metabolic, oxidative, and tissue-level effects of alendronate, standardized hop extract and their combination in an estrogen-deficient osteoporosis model. The integration of biochemical, histological, and lipidomic analyses allowed for assessment of early systemic responses to these interventions.

### 4.1. Oxidative Stress

Estrogen deficiency is often reported as a well-established contributor to oxidative stress, influencing bone turnover and promoting postmenopausal metabolic changes, as well as contributing to systemic damage, affecting liver, kidney, and cardiovascular tissues [69,70,71]. Contrary to expectations, ovariectomy did not significantly alter most markers of oxidative stress, including serum nitrites, MDA levels, and liver protein carbonyls. However, a reduction in catalase activity was observed in liver tissue of ovariectomized animals compared to controls, indicating a localized alteration in antioxidant defense. This suggests that estrogen deficiency may affect specific enzymatic components of the antioxidant system without inducing a generalized oxidative stress response within the timeframe studied.

Our study found no evidence of altered NO or arginase metabolism in the serum following the 30-day post-ovariectomy period and the short-term (2-week) treatment. Although alterations in NO bioavailability have been reported in estrogen-deficient settings [72,73,74], such effects were not observed here. Nevertheless, it can take months post-ovariectomy before significant changes in serum NO levels are detected [73].

No significant changes in MDA levels in liver and kidney indicate no measurable increase in lipid peroxidation caused by interventions during the study period. Previous studies have reported both increased lipid peroxidation following alendronate treatment and reduced oxidative stress with phytoestrogens in ovariectomized models [75,76,77]; however, such effects were not observed in the present study and may be model- and context-dependent.

Protein carbonyls were unchanged in the liver, and in kidney they were reduced by high-dose alendronate, indicating a localized effect on protein oxidation. This reduction was not evident in the combined treatment groups, suggesting that the effect may be attenuated in the presence of hop extract.

Catalase activity in liver was decreased following ovariectomy and increased by alendronate treatment, particularly at higher doses. This is in line with previous reports of alendronate-induced increases in catalase activity in experimental models, although in the present study this effect was limited to liver tissue [75,78]. The absence of this effect in combined treatment groups suggests that co-administration with hop extract may attenuate this response.

Overall, oxidative stress markers were largely unchanged across tissues, with only selective effects of alendronate on liver catalase activity and kidney protein carbonyls. Although interactions between alendronate and hop extract were detected, their biological significance remains unclear, and further studies are required to determine whether these effects persist under longer treatment conditions. It should also be taken into account that oxidative stress responses to estrogen deficiency may be time-dependent and partially compensated by adaptive cellular mechanisms [79,80].

### 4.2. Cholesterol and Triglycerides in Serum and Liver

Serum triglyceride levels did not differ significantly between groups, whereas total cholesterol was significantly increased in animals receiving hop extract. Two-way ANOVA confirmed a strong main effect of hop extract on serum cholesterol, with no effect of ovariectomy or alendronate, indicating that this change is treatment-specific. Nevertheless, based on the reference values for rat serum lipids, the measured cholesterol values are within normal ranges for these animals [81], although they can vary by strain, age, and diet. Therefore, the biological relevance of this serum cholesterol increase remains unclear, particularly in the absence of specific lipoprotein measurement (HDL vs. LDL).

Hepatic cholesterol levels were unchanged across groups, while triglyceride accumulation was the highest in untreated OV animals; however, these did not differ significantly from controls. Two-way ANOVA revealed a significant main effect of alendronate and a treatment interaction, indicating that reductions in hepatic triglycerides were primarily associated with alendronate and modified in combination with hop extract. These findings suggest modulation of hepatic lipid metabolism rather than reversal of ovariectomy-induced changes. Similar changes in hepatic triglycerides have been reported in experimental models following treatment with phytoestrogens and bisphosphonates [82,83].

### 4.3. Liver and Perigonadal Adipose Tissue Histology 

Histological analysis of liver tissue revealed no significant adverse changes in tissue architecture, fibrosis, or accumulation of connective tissue. Due to the relatively short experimental period, potential long-term effects cannot be excluded, as fibrosis induction requires a longer time in rodent models [84]. This warrants subsequent studies to investigate the long-term hepatic safety of the treatment.

Ovariectomy in rats is known to induce visceral fat accumulation, lipogenesis, and a pro-inflammatory state, often accompanied by hyperleptinemia and insulin resistance [85,86,87]. In this study, however, no significant differences in adipocyte morphology were observed between control and ovariectomized animals. Two-way ANOVA indicated a main effect of alendronate on adipocyte size and number, suggesting treatment-related modulation of adipocyte characteristics. Pairwise analysis showed that the X group had smaller adipocyte area and diameter, and a higher number of adipocytes per area compared to several treated groups; however, the results did not differ from OV or control animals. This indicates that these changes do not reflect reversal of ovariectomy-induced adiposity. Previous studies have reported that ovariectomy-induced adiposity can be modified by estradiol or phytoestrogens [87,88,89,90], although such effects were not observed in the present model.

Overall, neither hop extract nor alendronate induced adverse morphological changes in the liver or adipose tissue under the conditions of this study. As ovariectomy alone did not significantly alter these parameters, the observed differences likely reflect treatment-related metabolic modulation.

### 4.4. MALDI-TOF MS Analysis of Liver Metabolism

MALDI-TOF MS analysis demonstrated that ovariectomy did not induce significant changes in hepatic metabolism; however, the subsequent treatment with hop extract, alendronate, or their combination modulated different metabolic pathways in the liver, including amino acid, lipid, steroid, and redox-related metabolism.

The observed changes in amino acid metabolism (L-histidine and L-Argininosuccinate) suggest a possible impact of alendronate on histidine turnover and urea cycle, which is modified in the presence of hop extract. Deoxyuridine monophosphate (dUMP) showed bidirectional changes, indicating treatment-specific effects on nucleotide metabolism. While dUMP itself is primarily associated with DNA synthesis pathways, hepatic nucleotide pools more broadly are involved in conjugation reactions important for metabolite processing and biliary excretion, so these findings may reflect more general changes in hepatic nucleotide metabolism. Although no direct evidence of hop-derived flavonoids on thymidylate synthase can be found, some plant-derived flavonoids like quercetin and apigenin have been reported to influence enzymes involved in pyrimidine synthesis in cancer models [91,92]. The reduction in dUMP levels with a high alendronate dose was altered in the presence of hop extract.

Changes in fatty acids and their metabolites may reflect altered hepatic lipid handling [93]. Despite these molecular changes, hepatic triglyceride content and histological architecture remained unchanged, consistent with the absence of liver injury. Alterations in 2,3-Bis(O-phytanyl)-sn-glycerol-1-phosphate suggest alterations in specific glycerophospholipid-related intermediates in a treatment-specific manner.

Ophthalmate, a marker of oxidative stress, suggests a treatment-dependent modulation of hepatic redox state. Alendronate has been reported to influence oxidative stress parameters in other tissues [94], although conventional oxidative stress assays did not fully corroborate these changes. The increase in thyroxine (T4) suggests treatment-related modulation of hormone handling and is consistent with the liver’s central role in thyroid hormone metabolism and transport [95], but functional consequences cannot be inferred without targeted endocrine measurements.

These findings suggest adaptive, rather than pathological, metabolic responses and provide a basis for future mechanistic studies addressing long-term hepatic effects and systemic metabolic integration.

### 4.5. MALDI-TOF MS Analysis of Perigonadal Adipose Tissue

Changes in L-palmitoylcarnitine suggest the modulation of fatty acid transport into mitochondria and potentially of β-oxidation. Although no direct link between alendronate and increased L-palmitoylcarnitine is established, it is of note that alendronate acts via downregulation of the mevalonate pathway, which may secondarily influence mitochondrial function and lipid oxidation pathways.

Multiple long-chain fatty acids were elevated in the X group. These point to alterations in long-chain fatty acid composition in visceral adipose tissue. Interestingly, as discussed above, hop extract also reduced adipocyte size. These two findings in hop-treated animals may indicate altered lipid turnover or fatty acid handling [32,96]. However, only eicosenoic acid (C20:1) remained significantly altered when comparing treatments to ovariectomy. This indicates that most differences in fatty acids reflect differences relative to homeostatic state and not amelioration of ovariectomy impact.

Sphingolipid metabolites (in this case, sphinganine-1-phosphate) play different roles in adipocyte metabolism and cellular signaling [97], which may reflect adaptive remodeling rather than pathological accumulation. The putative effect of a high alendronate dose is yet to be investigated in this context.

Steroid-related metabolites were selectively affected in AH. These changes suggest the modulation of local sterol and estrogen conjugation pathways in adipose tissue, potentially reflecting altered steroid handling rather than systemic endocrine disruption.

The most pronounced differences relative to the control group were observed in the X group and primarily involved long-chain fatty acids. In contrast, the AH group demonstrated selective modulation of fatty acyl transport (L-palmitoylcarnitine), sphingolipid signaling (sphinganine-1-phosphate), and steroid derivatives. Compared to the non-treated ovariectomized group, there were fewer changes, indicating that many treatment-associated changes reflect shifts relative to basal physiological conditions.

Collectively, the groups that had significant metabolite changes both in liver and adipose tissue overlapped with groups showing effects on bone quality in the same experimental model [48], although these relationships require further investigation. Given the untargeted nature of the analysis, further clarification of these interactions is necessary by employing MS/MS analysis and further confirmation of observed changes (mostly in fatty acid metabolism) using additional methods.

### 4.6. Overall Interpretation and Implications

Taken together, this multi-level analysis shows that alendronate and hop extract exert distinct and independent effects on selected metabolic and oxidative parameters in this model. Alendronate was associated with changes in liver catalase activity, kidney protein carbonyls, and hepatic triglyceride levels, whereas hop extract showed a consistent effect on serum cholesterol. However, most measured parameters, particularly those related to oxidative stress, were not significantly affected by ovariectomy or treatment.

Importantly, the absence of consistent differences between control and ovariectomized animals for several outcomes suggests that these changes do not represent reversal of disease-related alterations. Interaction effects observed between alendronate and hop extract in some parameters should be considered in future translational studies.

MALDI-TOF MS analysis further indicated treatment-associated changes in hepatic and adipose tissue metabolite profiles; however, these were not consistently aligned with biochemical or histological outcomes and should be considered exploratory and interpreted in the context of the short treatment duration.

### 4.7. Study Limitations and Future Perspectives

This study has several limitations. Regarding the housing design, animals were group-housed in two cages per treatment group (as explained in the Methods); therefore, cage effect cannot be excluded. The individual animal was used as the experimental unit for statistics; this approach may overestimate independence between observations. In spite of the exploratory nature of the study and the generally modest effects observed across multiple outcomes, this limitation should be considered when interpreting the results. Next, the 2-week experimental treatment period was insufficient to evaluate long-term metabolic outcomes, such as fibrosis, advanced adipose dysfunction and similar effects. The results primarily reflect early responses to the compounds studied. Also, the absence of HDL/LDL quantification limits interpretation of the cholesterol changes observed in hop-treated animals. Metabolomic profiling was restricted to liver and visceral adipose tissue, without parallel transcriptomic or endocrine characterization. Finally, the exploratory design is also a limitation. Although adjustments were applied for the MALDI-TOF MS data, other analyses were not corrected for multiple comparisons and should be interpreted as hypothesis-generating rather than confirmatory. Future studies are needed to validate these findings.

Future long-term studies should include lipoprotein subfraction analysis and dose–dependent comparisons of standardized hop extract and/or isolated 8-prenylnaringenin. To further confirm metabolomic changes and metabolite identification, confirmation of key identified components with MS/MS analysis is necessary. Longitudinal studies of combination therapy with extended duration and follow-up, dynamic bone turnover markers and functional bone strength testing would also clarify the translational potential of this combined approach.

Overall, the present findings provide an exploratory assessment of the metabolic and tissue-level effects of alendronate and hop extract combination therapy in an estrogen-deficient model, complementing our findings on bone-related outcomes from the same research project, reported separately [48], most notably the effect of hop extract and combinations with alendronate. Additional tissue-specific outcomes from the same experimental cohort, including reproductive tissue analysis, have been reported previously [98]. Together, these datasets provide a more comprehensive understanding of the effects of hop-derived phytochemicals and their combinations with standardized osteoporosis treatment. While treatment-associated changes were observed across several parameters, their biological relevance and potential translational implications remain to be investigated. Further studies incorporating longer treatment durations, targeted metabolic profiling, and functional endpoints are needed to determine whether such combination therapy has potential in therapeutic use and how it affects long-term metabolic outcomes.

## Figures and Tables

**Table 1:** Summary report of two-way ANOVA analysis for selected parameters. Data are presented as *p*-values. Only statistically significant effects (*p* < 0.05) are shown; blank cells indicate non-significant effects. Arrows indicate direction of significant effects.

Parameter	Alendronate (*p*)	Hop Extract (*p*)	Interaction (*p*)	Interpretation
Serum total cholesterol	–	<0.001	–	↑ with hop extract
Protein carbonyls (kidney)	0.011	–	0.042	↓ with alendronate; interaction present
Catalase activity (liver)	<0.001	–	<0.001	↑ with alendronate; interaction present
Total triglycerides (liver)	0.03	–	0.004	↓ with alendronate; interaction present
Adipocyte area	0.008	–	–	↓ with alendronate
Adipocyte diameter	0.007	–	–	↓ with alendronate
Adipocyte number/area	0.007	–	–	↑ with alendronate

**Table 2:** Significant metabolite changes in liver tissue obtained by MALDI-TOF MS analysis and identified in the KEGG database.

	Identifications			Experimental Group (Adjusted *p* Value (*t*-Statistics)) ^†^					
RG *	KEGG ID	*m*/*z*	Tentative Annotation	OV	AL	AH	AL-X	AH-X	X
C	C00135	214.1	L-Histidine				0.028 (4.045)		
	C00365	307	Deoxyuridine monophosphate (dUMP)			0.029 (−4.197)			0.035 (4.197)
	C03406	308.2	L-Argininosuccinate			0.019 (−4.413)	0.046 (−4.322)		0.035 (4.002)
	C01571	231.2	Decanoic acid			0.009 (−7.024)	0.007 (−6.085)		0.004 (7.024)
	C00249	257.2	Palmitic acid						0.047 (3.559)
	C01530	283.3	Stearic acid			0.009 (5.862)			
	C20518	791.7	2,3-Bis(O-phytanyl)-sn-glycerol-1-phosphate			0.009 (−4.498)			0.004 (5.578)
	C21016	307.2	Ophthalmate			0.027 (−4.062)	0.046 (−4.051)		0.035 (3.910)
	C01829	835.7	Thyroxine (T4)						0.047 (3.166)
OV	C01571	231.2	Decanoic acid			0.034 (−5.078)			0.033 (−5.078)
	C00249	257.2	Palmitic acid						0.036 (−4.267)
	C01530	283.3	Stearic acid			0.030 (5.862)			
	C20518	791.7	2,3-Bis(O-phytanyl)-sn-glycerol-1-phosphate						0.033 (−4.012)

* Abbreviations: RG—reference group (with which the experimental groups were compared); C—sham-operated group; OV—untreated ovariectomized group; AL—ovariectomized low-alendronate group; AH—ovariectomized high-alendronate group; AL-X—ovariectomized low-alendronate and hop extract group; AH-X—ovariectomized high-alendronate and hop extract group; X—ovariectomized animals, hop extract group. ^†^ Results of Student’s *t*-test (comparison of experimental groups with reference groups).

**Table 3:** Significant metabolite changes in perigonadal adipose tissue obtained by MALDI-TOF MS analysis and identified in the KEGG database.

	Identifications			Experimental Group (Adjusted *p* Value (*t*-Statistics)) ^†^					
RG *	KEGG ID	*m*/*z*	Tenative Annotation	OV	AL	AH	AL-X	AH-X	X
C	C02990	438.3	L-Palmitoylcarnitine			0.010 (5.782)			
	C06425	293.3	Arachidic acid (C20:0)			0.010 (−5.050)			0.007 (5.809)
	C16526	293.3	Eicosenoic acid (C20:1)						0.007 (6.604)
	C08316	356.4	Erucic acid (C22:1)						0.030 (4.549)
	C08281	358.4	Behenic acid (C22:0)						0.025 (4.899)
	C08323	367.4	Nervonic acid (C24:1)						0.030 (3.970)
	C16527	313.3	Adrenic acid (C22:4)						0.007 (5.837)
	C01120	404.3	Sphinganine-1-phosphate			0.030 (4.747)			
	C05452	405.4	3α,7α-Dihydroxy-5β-cholestane			0.010 (−4.757)			
	C11133	469.2	Estrone-3-glucuronide			0.040 (4.385)			
OV	C02990	438.3	L-Palmitoylcarnitine			0.030 (5.782)			
	C16526	293.3	Eicosenoic acid (C20:1)						0.020 (−6.162)

* Abbreviations: RG—reference group (with which the experimental groups were compared); C—sham-operated group; OV—untreated ovariectomized group; AL—ovariectomized low-alendronate group; AH—ovariectomized high-alendronate group; AL-X—ovariectomized low-alendronate and hop extract group; AH-X—ovariectomized high-alendronate and hop extract group; X—ovariectomized animals, hop extract group. ^†^ Results of Student’s *t*-test (comparison of experimental groups with reference groups).

## Data Availability

The original contributions presented in this study are included in the article. Further inquiries can be directed to the corresponding author.
